# Spatial boundary of urban ‘acid islands’ in southern China

**DOI:** 10.1038/srep12625

**Published:** 2015-07-27

**Authors:** E. Du, W. de Vries, X. Liu, J. Fang, J. N. Galloway, Y. Jiang

**Affiliations:** 1 State Key Laboratory of Earth Surface Processes and Resource Ecology, and College of Resources Science & Technology, Beijing Normal University, Beijing, 100875, China; 2 Environmental Systems Analysis Group, Wageningen University, PO Box 47, 6700 AA Wageningen, the Netherlands; 3 Alterra, Wageningen University and Research Center, PO Box 47, 6700 AA Wageningen, the Netherlands; 4 College of Resources and Environmental Sciences, China Agricultural University, Beijing, 100193, China; 5 Department of Ecology, and Key Laboratory for Earth Surface Processes of the Ministry of Education, Peking University, Beijing, 100871, China; 6 Department of Environmental Sciences, University of Virginia, Charlottesville, VA 22904, USA

## Abstract

Elevated emissions of sulfur dioxide, nitrogen oxides and ammonia in China have resulted in high levels of sulfur and nitrogen deposition, being contributors to soil acidification, especially in and near large cities. However, knowledge gaps still exist in the way that large cities shape spatial patterns of acid deposition. Here, we assessed the patterns of pH, sulfate, nitrate and ammonium in bulk precipitation and throughfall in southern China’s forests by synthesizing data from published literature. Concentrations and fluxes of sulfate, nitrate and ammonium in bulk precipitation and throughfall exhibited a power-law increase with a closer distance to the nearest large cities, and accordingly pH showed a logarithmic decline. Our findings indicate the occurrence of urban ‘acid islands’ with a critical radius of approximately 70 km in southern China, receiving potential acid loads of more than 2 keq ha^−1^ yr^−1^. These urban acid islands covered an area of 0.70 million km^2^, accounting for nearly 30% of the land area in southern China. Despite a significant capacity to neutralize acids in precipitation, our analysis highlights a substantial contribution of ammonium to potential acid load. Our results suggest a joint control on emissions of multiple acid precursors from urban areas in southern China.

Elevated emissions of sulfur dioxide (SO_2_) and nitrogen oxides (NO_x_) have occurred during rapid industrialization and urbanization in China, especially in and near large cities^1^,^2^,^3^. Widespread areas in China, especially the southern regions, have been receiving considerable acid deposition^4^, which is likely to result in some key environmental issues, including acidification of terrestrial and aquatic ecosystems^4^,^5^,^6^, loss of biodiversity^6^, and damage of calcareous (e.g. concrete and marble) buildings and metal materials^7^. Although efforts have been made to improve the understanding of the status and effects of acid deposition in China^4^,^6^, knowledge gaps still exist in the way that large cities shape the spatial pattern of acid deposition.

Large cities with high population density are hotspots of anthropogenic emissions of acid precursors due to a combination of intensive energy production, motor traffic, waste treatment and industrial activities^1^,^8^,^9^. Concentrations and fluxes of nitrate (NO_3_^−^) in bulk precipitation have been found to exhibit a power-law increase with a closer distance to the center of the nearest large cities^10^. Because large cities also are hotspots of SO_2_ emissions, we hypothesized that sulfuric acid would behave the same way as nitrate. Assuming a proportional change in the neutralizing effect due to base cations and ammonia (NH_3_), we expected that hydrogen ion (H^+^) would also increase significantly the closer one gets to large cities. Based on the analysis above, we introduced a concept of urban ‘acid islands’, which implies that acid deposition increases with a closer distance to large cities. Therefore, the pH, defined as the negative logarithm of the activity of the hydrogen ion, is expected to show a logarithmic decline with a closer distance to large cities.

Although ammonia can neutralize sulfuric and nitric acids in cloud water and precipitation, subsequent ammonium (NH_4_^+^) deposition has a potential to generate significant acidification^11^. This is due to nitrification, causing the production of two protons (NH_4_^+^ + 2O_2_ → NO_3_^−^ + H_2_O + 2H^+^). Note that one proton is due to ammonia deposition, while the other proton comes from acids (e.g. sulfuric acid and nitric acid) in precipitation, being neutralized in the atmosphere (NH_3_ + H^+^ → NH_4_^+^). The potential acid load thus equals the sum of the deposition of the hydrogen ion and twice the ammonium ion (H^+^ + 2 NH_4_^+^), being the value with which critical acid loads have been compared since several decades^12^. Due to a rapid increase in NH_3_ emission^3^, high levels of ammonium deposition have been observed in China’s forests, being more than twice of nitrate deposition^10^. Therefore, it is essential to include ammonium when testing the hypothesis of urban acid islands. Apart from acidifying effects, increased loadings of nutrient nitrogen in ecosystems have the potential to release nitrogen limitation or enhance eutrophication, either resulting in an increase in net primary production or leading to nutrient imbalance and biodiversity loss^6^.

Anthropogenic emissions of acid precursors (SO_2_, NO_x_ and NH_3_) generally are low from forests and therefore concentrations and fluxes of sulfate, nitrate and ammonium in bulk precipitation at forested sites well indicate the ambient status of acid deposition. In northern China, acid deposition rarely become problematic due to neutralization by high levels of base cation deposition^13^, while acid deposition in southern China can lead to significant acidification^14^,^15^. Therefore, we focused our study on southern China (Fig. 1). Here, we tested the hypothesis of urban acid islands by synthesizing data from published literature on pH, nitrate, sulfate and ammonium in bulk precipitation and throughfall in southern China’s forests. Throughfall is a sum of bulk deposition, canopy captured dry deposition and canopy exchange^16^,^17^. Previous evidence indicates that throughfall deposition is a reasonable estimate of total adeposition^10^,^18^, although it may underestimate acid deposition due to canopy uptake of nitrogen compounds and hydrogen ion^19^,^20^,^21^. We then estimated the critical radius of the urban acid islands, based on a maximum critical load of acidification for soils in southern China, and discussed the policy implications for a better control of acid deposition in China.

## Results

### Concentrations and deposition of hydrogen ion, sulfate, nitrate and ammonium

Concentrations and fluxes of hydrogen ion, sulfate, nitrate and ammonium in bulk precipitation and throughfall generally were high in southern China, but large spatial heterogeneity existed (Figs 2, 3, 4, 5). Highest levels of acid deposition occurred around several hotspots, including Yangtze River delta, Changsha-Zhuzhou-Xiangtan city cluster, Wuhan city cluster, Chengdu-Chongqing city cluster, and Pearl River delta.

Values of pH in bulk precipitation showed a geometric mean of 5.5 (n = 28) and increased to 5.8 in throughfall. Geometric mean concentrations of sulfate, nitrate and ammonium in bulk precipitation were estimated at 101.2 (n = 30), 19.1 (n = 33) and 47.6 (n = 33) μeq L^−1^, respectively. Bulk deposition of sulfate, nitrate and ammonium showed geometric means at 1.55 (n = 30), 0.30 (n = 33) and 0.73 (n = 33) keq ha^−1^ yr^−1^, respectively. Throughfall concentrations of sulfate, nitrate and ammonium showed elevated geometric means at 184.2, 30.1 and 64.2 μeq L^−1^, leading to throughfall deposition of sulfate, nitrate and ammonium at 2.83, 0.47 and 0.98 keq ha^−1^ yr^−1^, respectively.

### Testing the hypothesis of urban acid islands

Values of pH in bulk precipitation showed a logarithmic decline with a closer distance to the center of the nearest large cities (Fig. 6a). Accordingly, concentrations of sulfate, nitrate and ammonium showed a power-law increase with a closer distance to the nearest large cities (Fig. 6b–d). Moreover, bulk deposition of hydrogen ion, sulfate, nitrate and ammonium all showed a power-law increase with a closer distance to the nearest large cities (Fig. 6e–h). Concentrations and fluxes of hydrogen ion, sulfate, nitrate and ammonium in throughfall behaved similar as those in bulk precipitation (Fig. 7).

### Estimating the critical radius of urban acid islands

Based on a critical acid load at 2 keq ha^−1^ yr^−1^ and an assessment of the potential acid load using the empirical equations on throughfall deposition of hydrogen ion and ammonium (Fig. 7e,h), we estimated a critical radius at 67 km for the urban acid islands in southern China. We further estimated that the total area of these urban acid islands was approximately 0.70 million km^2^ (Fig. 8), accounting for 29% of the land area in southern China.

## Discussion

In line with the hypothesis of urban acid islands, our results demonstrate a power-law increase of concentrations and fluxes of hydrogen ion, sulfate, nitrate and ammonium in bulk precipitation and throughfall with a closer distance to urban hotspots. Accordingly, pH values showed a logarithmic decline with a closer distance to large cities, which was consistent with a power-law increase of the hydrogen ion. In comparison with sulfate and ammonium, the less significant effect of urban areas on nitrate concentrations and nitrate deposition (Figs 6 and 7) is most likely caused by an intermixing effect of road networks^10^. Well-developed road network systems have severely fragmented the landscape in China^22^ and automobile NO_x_ emissions can substantially increase NO_x_ concentrations near roadside areas^23^, resulting in an intermixing effect of road networks on spatial patterns of nitrate deposition. Nevertheless, the concept of urban acid islands is an important approach to describe the way in which large cities shape the spatial pattern of acid deposition.

Uncertainties might exist in our analysis due to the close proximity of several cities in strongly urbanized regions, which could simultaneously contribute to the levels of acid deposition at a specific site. In that case, a larger critical radius would be expected. Here, we defined all the cities with nonagricultural population > 0.5 million as ‘large cities’ and this might also lead to uncertainties. Larger cities, having a wider spatial extent of acid precursors in the atmosphere^8^, would result in larger urban acid islands. Furthermore, spatial extent of acid precursors in the atmosphere generally varies with wind direction and wind velocity^8^ and therefore the radius of the urban acid islands is likely shorter in the upwind direction than in the downwind direction. The heterogeneity in spatial patterns of base cation deposition^13^ may also lead to uncertainties. Overall, our analysis most likely underestimated the critical radius of the urban acid islands because throughfall deposition is less than total deposition, especially for nitrogen compounds, due to significant foliar uptake^19^,^21^. This is also evident from the ratio of throughfall deposition versus bulk deposition, which is much lower for nitrogen compounds (1.56 for nitrate and 1.34 for ammonium) than for sulfate (1.82). Therefore, further assessments are needed to integrating detailed datasets on these factors as discussed above.

Our analysis shows that acid deposition in southern China was dominated by sulfuric acid, which contributed five to six times that of nitric acid, as determined by a comparison of sulfate to nitrate in bulk precipitation and throughfall. National average emissions of SO_2_ (14.4 TgS yr^−1^)^2^ and NO_x_ (5.0 TgN yr^−1^)^3^ during the period 2000 to 2009 lead to a ratio of acidification capacity near 2.5, being half of the estimates in southern China. As indicated by ammonium in bulk precipitation and throughfall, NH_3_ roughly neutralized 35% of the total acidity caused by sulfuric and nitric acids, while national average emissions of NH_3_ during the 2000s (12.7 TgN yr^−1^)^3^ suggest a potential to naturalize 72% of the precipitation acidity due to SO_2_ and NO_x_ emissions. Without the neutralization capacity of NH_3_ in southern China, the pH in precipitation would be significantly lower especially near large cities.

Despite a neutralization capacity of NH_3_ in the atmosphere, deposited ammonium has a potential to generate substantial soil acidification. By comparing ammonium deposition with sulfate and nitrate deposition, the net acidification capacity of ammonium deposition was estimated to contribute 25% to deposition-induced soil acidification in southern China. Integrating the contribution of nitric acid (11%), nitrogen deposition generally accounted for 36% of deposition-induced potential soil acidification, while sulfur deposition made a much higher contribution (64%). However, emission assessments^2^,^3^ lead to a lower contribution of SO_2_ (42%) and higher contributions of NO_x_ (16%) and NH_3_ (42%) at national scale.

In recent decades, China has made considerable efforts in curbing the problem of acid deposition, such as shifting the coal-dominated energy structure, developing desulfurization technologies and adopting stricter vehicular emission standards^24^. Due to these efforts, SO_2_ emissions have been reduced continuously since the middle 2000s but currently are still above the levels of the late 1990s^2^,^25^. In contrast, anthropogenic emissions of NO_x_ have increased from 1.3 TgN yr^−1^ in 1980 to more than 6 TgN yr^−1^ in 2010 (Ref. 3), but fortunately the government has recently set goals to reduce NO_x_ emissions by 10% in 2015 against the 2010 levels (Twelfth Five-year Plan) (A full version of the plan is available at http://news.xinhuanet.com/politics/2011-03/16/c_121193916.htm). However, our analysis indicates that sulfuric acid still dominates the acid deposition in southern China, while the contribution of nitric acid is likely growing in importance due to the stricter control of SO_2_ emissions and dramatic increase in NO_x_ emissions^3^,^25^, showing a same trend as that in Europe and USA in the early 1980s^11^. In addition, NH_3_ emissions have been more than doubled since 1980s (from 5.6 to 14.5 Tg N yr^−1^)^3^ and need urgent regulation in view of the significant potential of ammonium deposition in soil acidification^11^. There will also be a co-benefit from a long-term target to reduce emissions of CO_2_ and other pollutants for China^24^,^26^.

On the basis of our analysis, urban acid islands with a radius of approximately 70 km in southern China (Fig. 8), receiving potential acid loads of more than 2 keq ha^−1^ yr^−1^, are at high risk to soil acidification. Most of these urban acid islands are located around several hotspots, including Yangtze River delta, Changsha-Zhuzhou-Xiangtan city cluster, Wuhan city cluster, Chengdu-Chongqing city cluster, and Pearl River delta. The occurrence of urban acid islands may significantly contribute to acidification of arable lands (as lime application is not common in China’s cropland), forests and water bodies in suburbs of large cities^5^,^27^,^28^, potentially threatening agricultural production, forest health and water quality. Since the late 1990s, control zones of SO_2_ emissions and acid deposition have been designed as the first-generation approach to set priority regions for acid-rain reduction^15^. Our findings suggest that a second-generation policy for the reduction of acid deposition in China should also focus on the urban acid islands.

In conclusion, our analysis indicates that large cities in southern China are ‘acid islands’. Concentrations and fluxes of sulfate, nitrate, hydrogen ion and ammonium in bulk precipitation and throughfall exhibited a power-law increase with a closer distance to large cities, and therefore the pH showed a logarithmic decline. The concept of urban acid islands is useful in predicting acid deposition at regional scales. Although SO_2_ has been controlled for two decades in China^15^, sulfur deposition still contributes largest to soil acidification in southern China. Despite a significant capacity of NH_3_ to neutralize acids in precipitation, high levels of ammonium deposition have a substantial contribution to soil acidification in southern China. Our results also have important implications for the control of acid deposition *via* a joint control on emissions of multiple acid precursors, including not only SO_2_ and NO_x_ but also NH_3_, especially in and near large cities.

## Methods

### Data set

**Figure 1:**
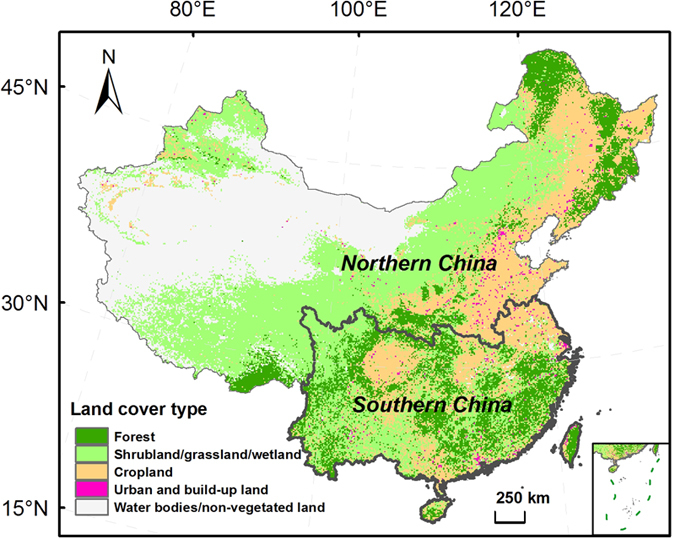
Map of northern and southern China with land cover information. Southern China covers a land area of 2.42 million km^2^ and holds approximately 60% of the national population. The map is generated by ArcGIS Desktop (version 9.3, ESRI, Redlands, USA).

**Figure 2:**
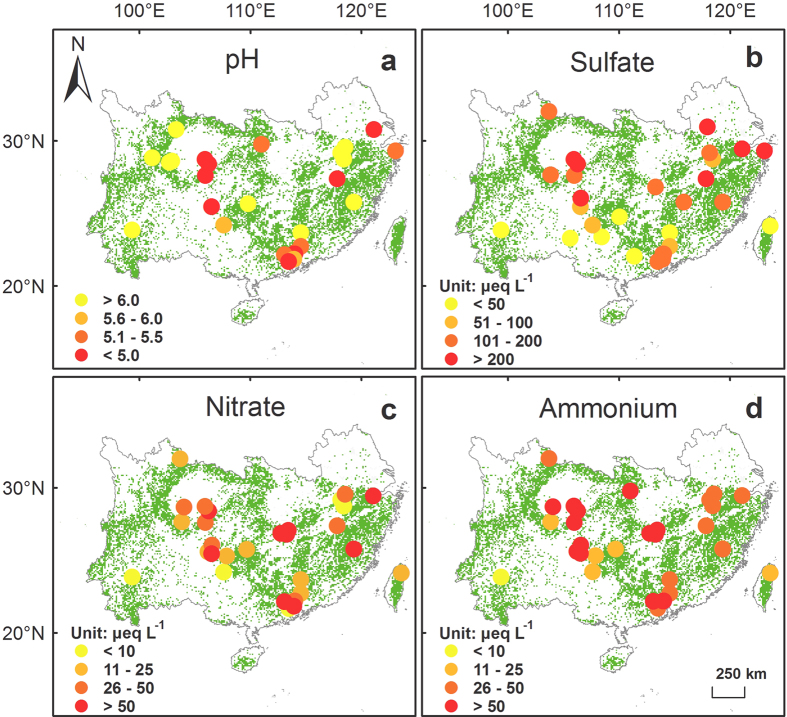
Spatial patterns of (**a**) pH, and concentrations (μeq L^−1^) of (**b**) sulfate, (**c**) nitrate, and (**d**) ammonium in bulk precipitation in southern China’s forests. Green background shows the distribution of forests. The figure is generated by ArcGIS Desktop (version 9.3, ESRI, Redlands, USA).

**Figure 3:**
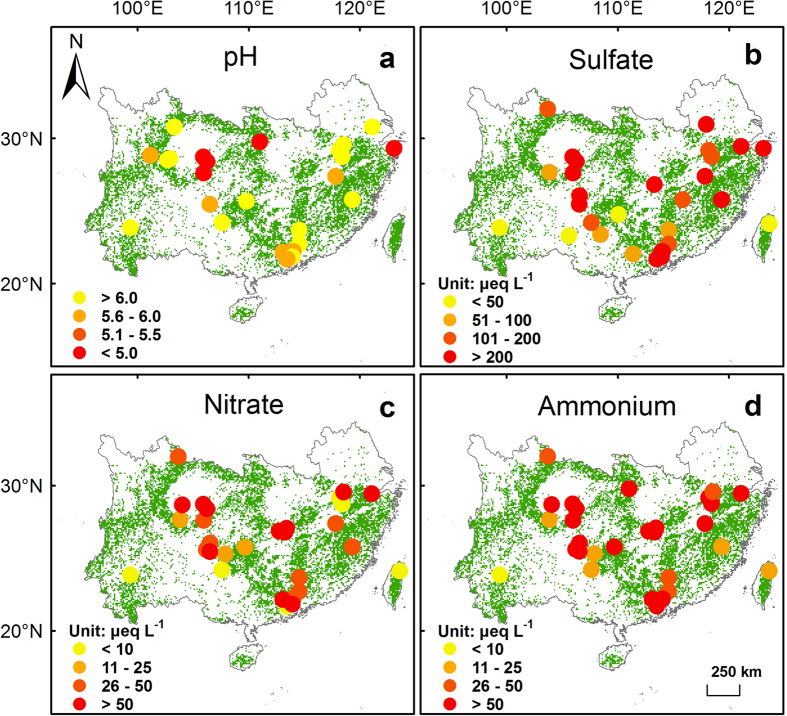
Spatial patterns of (**a**) pH, and concentrations (μeq L^−1^) of (**b**) sulfate, (**c**) nitrate, and (**d**) ammonium in throughfall in southern China’s forests. Green background shows the distribution of forests. The figure is generated by ArcGIS Desktop (version 9.3, ESRI, Redlands, USA).

**Figure 4:**
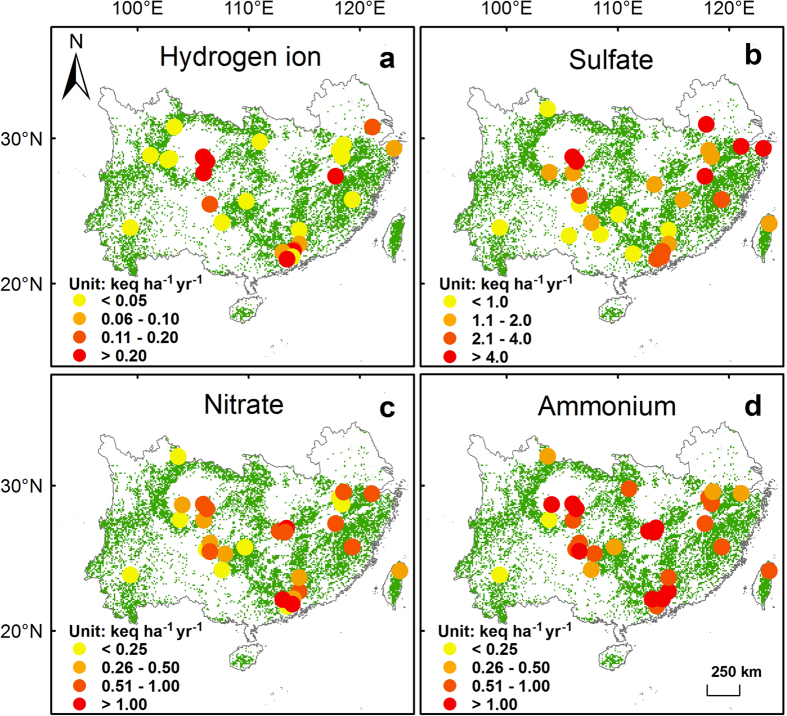
Bulk deposition (keq ha^−1^ yr^−1^) of (**a**) hydrogen ion, (**b**) sulfate, (**c**) nitrate and (**d**) ammonium in southern China’s forests. Green background shows the distribution of forests. The figure is generated by ArcGIS Desktop (version 9.3, ESRI, Redlands, USA).

**Figure 5:**
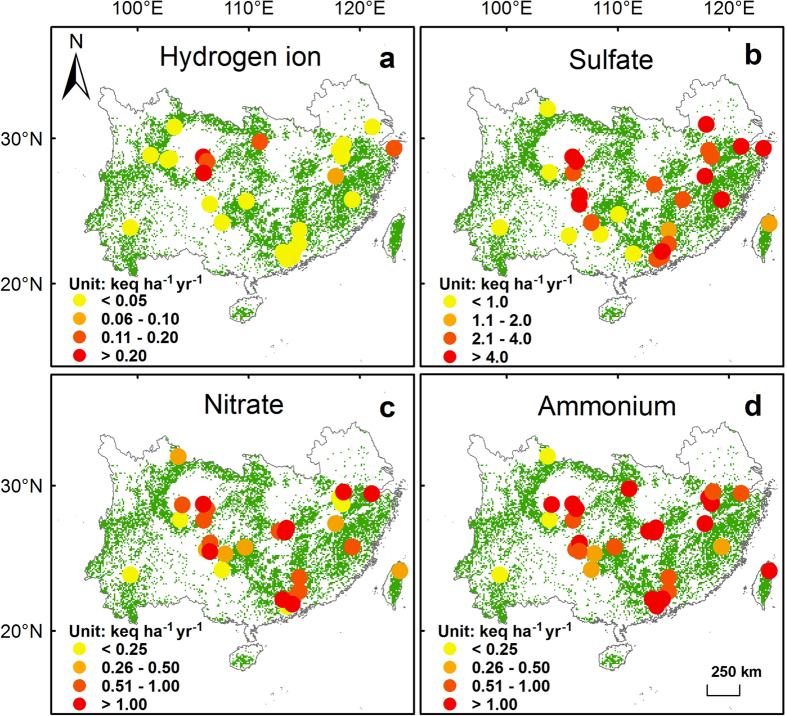
Throughfall deposition (keq ha^−1^ yr^−1^) of (**a**) hydrogen ion, (**b**) sulfate, (**c**) nitrate and (**d**) ammonium in southern China’s forests. Green background shows the distribution of forests. The figure is generated by ArcGIS Desktop (version 9.3, ESRI, Redlands, USA).

**Figure 6:**
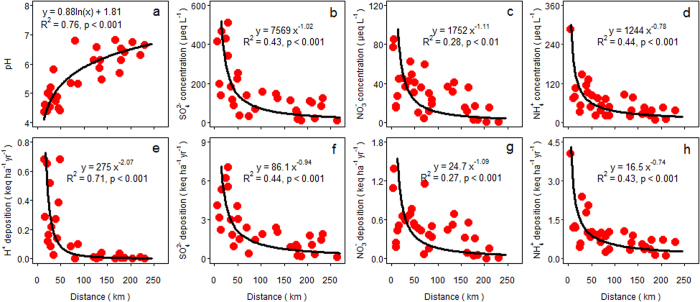
Changes in (**a**) pH, concentrations (μeq L^−1^) of (**b**) sulfate (SO_4_^2−^), (**c**) nitrate (NO_3_^−^) and (**d**) ammonium (NH_4_^+^) in bulk precipitation and bulk deposition (keq ha^−1^ yr^−1^) of (**e**) hydrogen ion (H^+^), (**f**) sulfate, (**g**) nitrate and (**h**) ammonium with a closer distance (km) to the center of the nearest large cities.

**Figure 7:**
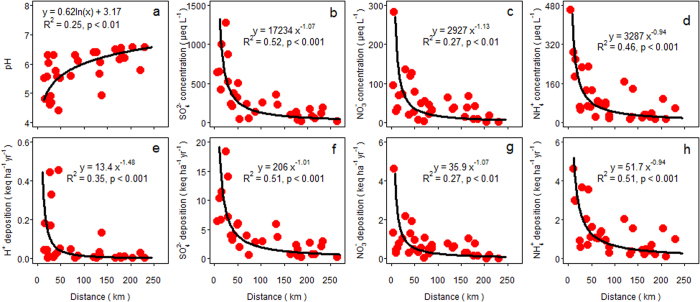
Changes in (**a**) pH, concentrations (μeq L^−1^) of (**b**) sulfate (SO_4_^2−^), (**c**) nitrate (NO_3_^−^) and (**d**) ammonium (NH_4_^+^) in throughfall and throughfall deposition (keq ha^−1^ yr^−1^) of (**e**) hydrogen ion (H^+^), (**f**) sulfate, (**g**) nitrate and (**h**) ammonium with a closer distance (km) to the center of the nearest large cities.

**Figure 8:**
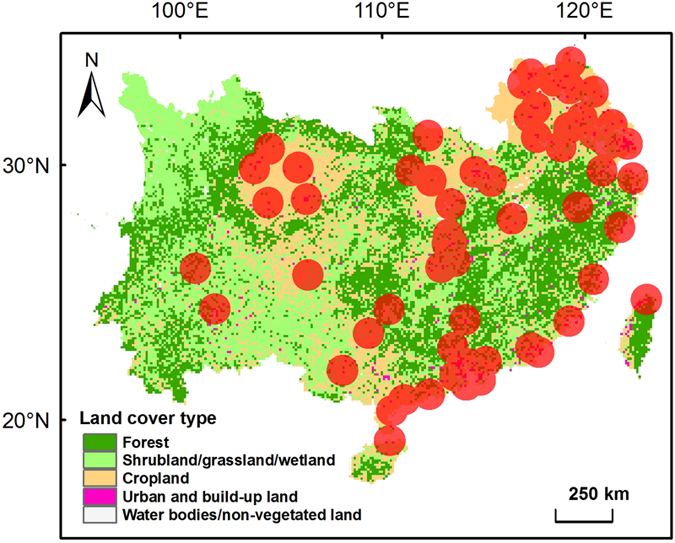
Distribution of urban acid islands in southern China. Large cities (nonagricultural population > 0.5 million) are illustrated as acid islands (in red, receiving potential acid loads of more than 2 keq ha^−1^ yr^−1^) with a critical radius of 67 km. The figure is generated by ArcGIS Desktop (version 9.3, ESRI, Redlands, USA).

The hypothesis of urban acid islands was tested by collecting data from published literature on pH and concentrations of sulfate, nitrate and ammonium in bulk precipitation and throughfall in typical forests in southern China (Fig. 1), as well as information on site location (latitude and longitude) and annual mean precipitation. Chemical data were selected only when precipitation and throughfall were measured simultaneously. Targeted data were either taken directly from tables or digitized from figures. Volume-weighted means of pH and concentrations of nitrate, sulfate and ammonium were calculated for the whole sampling period. Bulk and throughfall deposition was calculated based on the multiplication of annual precipitation and volume-weighted mean concentration. The distance between the sampling site and the center of the nearest large city (nonagricultural population > 0.5 million) was derived using Google Earth for Microsoft Windows (Version 7.1.2.2041, Google Inc., Mountain View, USA). Our database included information on pH for 28 sites, and on concentrations of sulfate, nitrate and ammonium for 30, 33 and 33 sites, respectively. Detailed information on each site, including annual mean precipitation, observation year(s) and the related references is given in the Supplementary Material (Table S1).

### Statistical analysis

We tested the hypothesis of urban acid island by assessing whether both concentrations and fluxes of hydrogen ion, sulfate, nitrate and ammonium in bulk precipitation and throughfall show a power-law increase with a closer distance to large cities, according to equation (1),


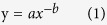


where y is volume-weighted mean concentration (μeq L^−1^) or annual flux (keq ha^−1^ yr^−1^), *x* is the distance (km) between the sampling site and the center of the nearest large city, a and b are parameters. In addition, we tested whether the value of pH shows a logarithmic decline with a closer distance to large cities, as described in equation (2),


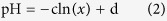


where *x* is the distance (km) between the sampling site and the center of the nearest large city, c and d are parameters.

In comparison with bulk deposition, throughfall deposition has been used as a more precise estimate of total deposition^10^,^18^. Based on a recent assessment^29^, critical loads of acidification for soils are always lower than 2 keq ha^−1^ yr^−1^ in southern China, except for some insensitive systems. Therefore, we estimated the critical distance of urban acid islands based on the empirical equations on the potential acid load (H^+^ + 2NH_4_^+^) via throughfall deposition and a maximum critical acid load at 2 keq ha^−1^ yr^−1^. All statistical analysis was performed using R software (version 3.1.0; R Development Core Team, 2014, http://www.r-project.org/) with a significance level of P < 0.05.

## Additional Information

**How to cite this article**: Du, E. *et al*. Spatial boundary of urban ‘acid islands’ in southern China. *Sci. Rep*. **5**, 12625; doi: 10.1038/srep12625 (2015).

## Supplementary Material

Supplementary Information
