# Supplementary material for: Spatial boundary of urban ‘acid islands’ in southern China
Source: Sci Rep. 2015 Jul 27;5:12625. doi: 10.1038/srep12625 (PMC4515822; doi:10.1038/srep12625)
Supplement: Supplementary Information [file srep12625-s1.pdf]

Spatial boundary of urban ‘acid islands’ in China

E. Du, W. de Vries, X. Liu, J. Fang, J. N Galloway, Y. Jiang

Table S1. Information on each site of the datasets.

Site ID	pH	SO_4^{2-}	NO_3^-	NH_4^+	AP (mm)	Year(s)	Reference
1	N	Y	Y	Y	805	2002-2003	Gong et al., 2005
2	Y	N	N	N	945	2006	Chang, 2007
3	N	Y	N	N	1000	2011	Zeng, 2012
4	N	Y	Y	Y	1022	2012-2013	Zhang et al., 2014
5	Y	Y	Y	Y	1100	2007-2008	Li et al., 2010
6	N	N	Y	Y	1120	2001-2003	Chen and Mulder, 2007
7	Y	Y	Y	Y	1145	1991-1992	Zhang et al., 1996
8	Y	N	N	Y	1150	2006	Wan et al., 2010
9	N	Y	Y	Y	1200	2001-2005	Zhang et al., 2007
10	Y	Y	Y	Y	1200	2004	Chen et al., 2009
11	Y	N	N	N	1200	2003	Song et al., 2005
12	N	N	Y	Y	1230	2001-2003	Chen and Mulder, 2007
13	N	Y	N	N	1248	1990-1991	Huang and Liang, 1994
14	N	N	Y	Y	1250	2001-2003	Chen and Mulder, 2007
15	Y	N	Y	Y	1300	1995-2002	Tian, 2002; Kang et al., 2006
16	Y	Y	N	N	1375	2009	Shen, 2011
17	N	N	Y	Y	1411	2007-2008	Luo and Wen, 2010
18	N	Y	Y	Y	1442	2009	Kong, 2010
19	N	Y	N	N	1538	1990-1991	Huang and Liang, 1994
20	N	Y	N	N	1539	2001-2002	Cai et al., 2003
21	N	N	Y	Y	1549	2003-2004	Chen et al., 2006
22	Y	N	Y	Y	1550	2008-2009	Zhang et al., 2010a
23	Y	Y	Y	Y	1550	2000-2002	Zhang et al., 2003&2007
24	N	Y	N	N	1582	1990-1991	Huang and Liang, 1994
25	Y	Y	Y	Y	1600	1998	Zhou et al., 2000
26	Y	Y	Y	Y	1600	1998-1999	Zhou et al., 2000
27	Y	Y	Y	Y	1612	2009-2012	Guo et al., 2012; Tang et al., 2013
28	Y	Y	Y	Y	1626	2011	Zeng, 2012; Zhang, 2010
29	Y	Y	Y	N	1629	2002-2003	Chen et al., 2004
30	Y	Y	Y	Y	1664	1994-1996	Fan and Hong, 2001
31	Y	Y	Y	Y	1664	1994-1996	Fan and Hong, 2001
32	Y	Y	Y	Y	1700	1996;2006-2007	Liu et al., 2000; Shen et al., 2013
33	Y	N	N	N	1700	2005	Xiao et al., 2010
34	N	N	Y	Y	1740	2001-2003	Chen and Mulder, 2007
35	Y	Y	Y	Y	1750	2011	Zhang, 2010a; Zeng, 2012
36	Y	Y	Y	Y	1752	2007-2008	Lu et al., 2010
37	N	Y	N	N	1779	1990-1991	Huang and Liang, 1994
38	Y	Y	Y	Y	1795	2009-2010	Wang and Wang, 2011
39	Y	Y	Y	Y	1900	2013	Li et al., 2014
40	Y	N	Y	Y	1929	1997-1999;2009	Zhang et al., 2010b
41	Y	Y	Y	Y	1931	1998-1999	Liu et al., 2002
42	Y	N	N	N	1947	2005-2006	Sun and Wang, 2009
43	Y	N	N	N	1949	2004	Tao et al., 2006
44	Y	Y	Y	Y	2144	2006-2008	Zhou et al., 2009
45	N	N	Y	Y	2210	2001-2003	Chen and Mulder, 2007
46	N	Y	Y	Y	4500	1994-1996	Lin et al., 2000

*Note: AP indicates annual mean precipitation. Y and N indicate the availability and unavailability of the targeted data, respectively.

References

Cai, T.J., Li, F., Li, J.Y., Chen, Y.R., Li, H.T., Zhang, H.Z. 2003. A study on rainfall chemistry of artificial forests in red earth hilly area. Journal of Natural Resources 18(1): 99–104.

Chang, Z.Y. 2007. Comparison on capability of water holding and nutrition preserving in artificial hybrid forest and second shrub-forest. Master dissertation. Southwest University.

Chen, B.F., Chen, Y., Ying, G.T., Ye, S.S., Ouyang, W., Lin, M.X. 2004. Study on the water quality of urban forest ecosystem in the Pearl River Delta. Forest Research 17(4): 453–460.

Chen, J., Meng, X.X., Zhang, W.D, Li, D.Q. 2009. Effects of forest canopy on precipitation chemical composition in Simian Mountain, Chongqing. Journal of Anhui Agricultural Sciences 37 (32), 16098–16101.

Chen, S.J., Tian, D.L., Yan, W.D., Xiang, W.H. 2006. Hydrochemical characteristics of throughfall in different layers of *Cinnamomum camphora* plantation. Chinese Journal of Ecology 25(7), 747–752.

Chen, X.Y., Mulder, J. 2007. Atmospheric deposition of nitrogen at five subtropical forested sites in South China. Science of the Total Environment 378, 317–330.

Fan, H.B., Hong, W. 2001. Estimation of dry deposition and canopy exchange in Chinese fir plantations. Forest Ecology and Management 147, 99–107.

Gong, H.D., Wang, K.Y., Yang, W.Q. 2005. Nutrient characteristics of throughfall and stemflow in three forests at the subalpine of western Sichuan. Scientia Silvae Sinicae 41(5), 14–20.

Guo, P., Wang, Y.Q., Wang, Y.J., Wang, R., Hu, B., Tang, X.F. 2012. Preliminary study of the chemical properties of broad-leaved forest under acid deposition in Jinyun Mountain. Journal of Soil and Water Conservation 26(5), 235–538.

Huang, C.B., Liang, H.W. 1994. Stemflow of main forest types in Guangxi subtropics. Journal of Plant Resources and Environment 3(4), 10–17.

Kang, W.X., Deng, X.W., Zhang, Z.H. 2006. Effects of canopy interception on water and nutrient cycling in the Chinese fir plantation ecosystem. Scientia Silvae Sinicae 42(12), 1–5.

Kong, W.J. 2010. Research on the hydrological effects of typical forest types in Miaoshanwu Nature Reserve. Master dissertation, Chinese Academy of Forestry.

Lang, Y. ,Cai, T.J., Chai, R.S., Su Y. 2012. Effects of different types of original *Pinus Korainensis* forest on precipitation hydro-chemical characteristics. Journal of Soil and Water Conservation 26(2), 184–191.

Li, J., Li, W., Hou, J.X., Gao, F. 2010. Typical forests' leaching characteristics during acid rain in Guizhou. China Environmental Science 30(10), 1297–1302.

Li, L., Liu, L.Q., Zhou, G.Y., Qiu, Z.J., Zhao, H.B. 2014. Effects of canopy damage on hydrochemistry of throughfall and stemflow in evergreen broadleaved forest of Nanling Moutains. Journal of Soil and Water Conservation 28(2): 45–68.

Lin, T.C., Hamburg, S.P., King, H.B., Hsia, Y.J. 2000. Throughfall patterns in a subtropical rainforest of northern Taiwan. Journal of Environment Quality 29, 1186–1193

Liu, J.X., Wen, D.Z., Zhou, G.Y. 2000. Chemical properties of the rainfall in the coniferous and broadleaved forests in acid rain area of Heshan, Guangdong. China Environmental Science 20(3), 198–202.

Liu, W.Y., Fox, J.E.D., Xu, Z.F. 2002. Nutrient fluxes in bulk precipitation, throughfall and stemflow in montane subtropical moist forest on Ailao Mountains in Yunnan, south-west China. Journal of Tropical Ecology 18, 527–548.

Lu, X.Q., Ding, F.J., Fang, S.Z., Toda, H., Haibara, K., Wei, L.M. 2010. Characteristics of nutrient elements with water transport in the primary forest in a Karst area of Guizhou Province. Acta Ecologica Sinica 30(20), 5448–5455.

Luo, Z., Wen, S.Z. 2010. Canopy interception and changes in nutrient concentrations in a *Liquidambar formosana* plantation inTianjiling forestry farm. Journal of Central South University of Forestry &Techmology 30(2), 55–59.

Mo, J.M., Fang, Y.T., Zhang, D.J., Kong, G.H., Feng, Z.N. 2002. Effects of rainfall reallocation on nutrient dynamic of a pine forest in Dinghushan. Guihaia 22(6), 529–533.

Shen, H.T. 2011. The eco-hydrological characteristics of the successional stages of evergreen broadleaved forests in Tiantong. Ph.D dissertation, East China Normal University.

Shen, W., Ren, H., Darrel Jenerette, G., Hui, D., Ren, H. 2013. Atmospheric deposition and canopy exchange of anions and cations in two plantation forests under acid rain influence. Atmospheric Environment 64, 242–250.

Song, Y.Z., Qin, B.Q., Yang, L.Y., Hu, W.P., Luo, L.C. 2005. Role of typical arbor layer in neutralizing acid rain in northern Taihu District. Journal of Lake Science 17(2), 157–161.

Sun, X.Y.,Wang, G.X. 2009. The hydro-chemical characteristics study of forest ecosystem precipitation distribution in Gongga Mountain. Research of Soil and Water Conservation 16(6), 120–124.

Tang, X.F., Wang, Y.Q., Wang, Y.J., Zhang, H.L., Guo, P., Hu, B. 2013. Canopy leaching characteristics of typical Forests during acid rain at Jinyun Mountains, Chongqing. Forest research 26(5), 548–553.

Tao, Y.P., Wu, N., Luo, P., Yi, S.L., Liu, B., Zhang, Q.Y., Liao, Y.Z. 2006. Study on the function of forest in filtering wet deposition of pollutants. Water Resources Protection 22(3), 16–23.

Tian, D.L. 2002. Nutrient contents in the rainfall inside and outside the stands of Guangping forest zone, Huitong. Journal of Central South Forestry University 22(3), 9–13.

Wan R., Wang, P.C., Zeng, L.X., Shi ,Y.H., Pan, L. 2010. Chemical properties of the precipitation circulation in the forest of Lanlingxi small watershed in the Three Gorges Reservoir Area. Journal of Nanjing Forestry University 34(3), 39–44.

Wang, W.M., Wang, X.X. 2011. Reallocation and chemical characteristics of rainfall in *Pinus massoniana* and *Schima superb* forests in subtropical China. Journal of Central South University of Forestry & Technology 31(9), 80–86.

Xiao, Y.H., Chen, B.F., Pan, Y.J., Shi, X., Xu, M. 2010. Effects of hydrological environment on tropical evergreen broad-leaved forest in Maofengshan Mountain of Guangzhou. Journal of Northeast Forestry University 38(3), 78–81.

Zeng, L.M. 2012. The characteristics of hydrochemistry of precipitation in subtropical urban and rural forest ecosystems in Anhui. Master dissertation, Anhui Agricultural University.

Zhang, F.Z., Zhang, J.Y., Zhang, H.R., Ogura, N., Ushikubo, A. 1996. Chemical composition of precipitation in a forest area of Chongqing, southwest China. Water, Air and Soil Pollution 90, 407–415.

Zhang, G., Zeng, G.M., Jiang, Y.M., Liu, H.L. 2003. The distributional characteristics of ions in the bulk precipitation and forested thoughfall in Shaoshan, Hunan Province. Research of Environmental Sciences 16(3), 14–17.

Zhang, G., Zeng, G.M., Du, C.Y., Jiang, Y.M., Su, X.K., Xiang, R.J., Huang, L., Xu, M., Zhang, C. 2007. Deposition patterns in bulk precipitation and throughfall in a subtropical mixed forest in central-south China. Forestry 80(2), 211–221.

Zhang, J., Liu, Y., Zhang, J., Ou, J., Cui, N.J. 2014. Rainfall redistribution by crown layer and variation characteristics of nitrogen and phosphorus in *Pinus massoniana* plantations. Chinese Journal of Ecology 33(6), 1451–1458.

Zhang, X., Xue, J.H., Kikuo, H., Xu, X.T., Tian, Y., Hiroto, T., Liu, Y.H. 2007. Nutrient dynamics and hydrological process of Karst forests in mountainous area of central Guizhou province, China. Journal of Plant Ecology 31(5), 757–768.

Zhang, Y.Q., 2010. The characteristics of hydrochemistry in subtropical urban and rural forest ecosystems in Anhui. Mater thesis. Anhui Agricultural University.

Zhang, Y.Q., Wang, L.J., Ding, Z.L., Zeng, L.M., Xu, X.N. 2010a. Dynamics of TOC and N in precipitation in subtropical evergreen broad-leaved forest at Laoshan Mountains, South Anhui. Journal of Soil and Water Conservation 24(2), 146–164.

Zhang, N., Qiao, Y.N., Liu, X.Z., Chu, G.W., Zhang, D.Q., Yan, J.H. 2010b. Nutrient characteristics in incident rainfall, throughfall, and stemflow in monsoon evergreen broad-leaved forest at Dinghushan. Journal of Tropical and Subtropical Botany 18, 502–510.

Zhou, G.Y., Xu, Y.G., Wu, Z.M., Luo, T.S., Li, B.Q., He, Z.C. 2000. Influences of acid rain on crown leaching of chemical ions in different forest ecosystems in Guangzhou. Forest Research 13(6), 598–607.

Zhou, G.Y., Tian, D.L., Qiu, Z.J., Deng, X.W., Wang, X., Liu, M. 2009. Crown effects on ion concentration in throughfall of a coniferous-broadleaved stand under acid deposition at Liuxihe, Guangzhou. Journal of Central South University of Forestry & Technology 5, 32–38.
